# Discovering the Unexpected with the Utilization of NGS in Diagnostics of Non-syndromic Hearing Loss Disorders: The Family Case of *ILDR1*-Dependent Hearing Loss Disorder

**DOI:** 10.3389/fgene.2017.00095

**Published:** 2017-06-30

**Authors:** Jernej Kovač, Gašper Klančar, Katarina Trebušak Podkrajšek, Saba Battelino

**Affiliations:** ^1^Unit of Special Laboratory Diagnostics, University Children’s Hospital, University Medical Centre LjubljanaLjubljana, Slovenia; ^2^Faculty of Medicine, University of LjubljanaLjubljana, Slovenia; ^3^Department of Otorhinolaryngology and Cervicofacial Surgery, University Medical Centre LjubljanaLjubljana, Slovenia

**Keywords:** ILDR1, hearing loss, cochlear implant, orphan disease, NGS analysis

## Abstract

Sensorineural hearing loss (SNHL) is a heterogeneous family of hearing disabilities with congenital (including genetic) as well as acquired etiology. Congenital SNHL of genetic etiology is further sub-divided into autosomal dominant, autosomal recessive and X-linked SNHL. More than 60 genes are involved in the etiology of autosomal recessive non-syndromic hearing loss (ARNSHL) commonly manifesting as heterogeneous pre-lingual profound to severe non-progressive clinical phenotype. *ILDR1*-dependent ARNSHL (DFNB42, OMIM: # 609646) is a very rare sub-type of hearing disability, with unknown prevalence, caused by function-damaging genetic variants in *ILDR1* gene reported in families of Middle-Eastern origin. *ILDR1* (Immunoglobulin-Like Domain-containing Receptor 1) is involved in the development of semicircular canal, tricellular tight junction and auditory hair cells. An apparently non-consanguineous family of European ancestry with two affected siblings with profound progressive hearing loss characterized in their infancy and successfully treated with cochlear implants (CI) is presented. Genetic analysis of common ARNSHL genetic causes in the population of origin was negative, thus the next-generation sequencing (NGS) and family segregation analysis to identify underlying causative genetic variant was performed. Unexpectedly and atypical for the population of origin a homozygous non-sense variant *ILDR1* c.942C > A (p.Cys314Ter) inherited from both heterozygous parents was identified in both patients. Contrary to the commonly reported phenotype, indices of a progressive hearing loss and potential compensatory mechanism of vestibular function were revealed with the analysis of clinical data. The utilization of NGS was demonstrated as an invaluable tool for the detection of atypical rare variants in diagnostics of unidentified hearing loss disorders.

## Case Presentation

A family case of hereditary hearing loss in two siblings of apparently non-consanguineous parents of European ancestry is presented. The evaluation protocol assessed detailed family history and medical history with focus on potential causes of acquired hearing loss (acoustic trauma, intrauterine infections, perinatal complications, meningitis, mumps, and prenatal/postnatal ototoxic drug exposure). Additionally, a complete audiological history was recorded to establish the age of the hearing loss onset, the rate of hearing loss progression and to identify other audiological symptoms. Both index patients and their parents underwent a clinical otorhinolaryngological examination, including ear microscopy, with a systematic search for syndromic hearing loss indices. A tympanogram was performed and middle-ear associated causes of hearing loss were excluded. Additionally, they underwent ophthalmological and pedo-neurological examinations. The conditional and partly conditioned classical pure tone audiogram (PTA) was recorded in speech frequencies 0.25, 0.5, 1, 2, and 4 kHz, respectively, followed by the brainstem evoked response audiometry (BERA) analysis with up to 110 dB click stimulation. The function of vestibular organs was tested with caloric stimulation by irrigation of the external ear canal with 50 cm^3^ of water at 44, 30, and 17°C, respectively. Before cochlear implant surgery (CI) the computed tomography (CT scan) of the temporal bone was performed to evaluate the anatomy of medial and inner ear. Additionally, cochleography and electrically evoked auditory brain responds (EABR) test by reversed electrical 200 μs long stimuli with intensity range from 200 μA to 1 mA, using stimulating golf electrode placed on a round window was performed. This study was carried out in accordance with the recommendations of the Declaration of Helsinki and the Slovenian national medical ethics committee. All subjects or their legal guardians gave written informed consent in accordance with the Declaration of Helsinki. The protocol was approved by the Slovenian national medical ethics committee (#34/4/07).

**FIGURE 1:**
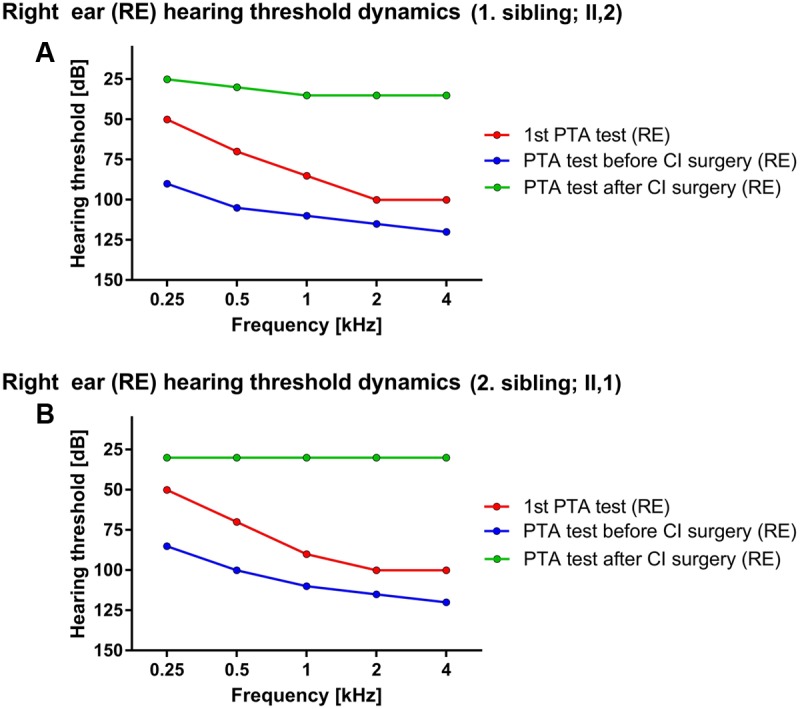
Hearing threshold dynamics of PTA test across analyzed frequencies (0.25–4 kHz). The prominent drop in lower frequency range was detected in both patients – red and blue lines in **(A)** (1. sibling) and **(B)** (2. sibling) with hearing most severely affected at higher frequencies, thus forming a characteristic PTA curve for *ILRD1*-dependent hearing loss. The CI surgery significantly improved the hearing across whole frequency range (green line).

First sibling (II,2; age range: 35–40 years) was admitted for the first time to an audiologist (age range: 0–5 years) due to hearing disorder and fitted with bilateral hearing aids. The caloric stimulation of both vestibular organs was negative, yet the patient did not present with any vestibular problems in recorded medical history. Interestingly, the speech was well developed, enabling education by regular educational program. The hearing above 3 kHz rapidly deteriorated, followed by relatively stable period until the adulthood (age range: 30–35 years) when only some islands of residual hearing were detected at lower frequencies. The level of hearing perception on PTA test performed at the first audiologist examination were 50, 70, 85, 100, and 100 dB for the right side and 40, 55, 70, 90, and 90 dB for left side, respectively. Later the hearing significantly worsened with PTA thresholds reaching 90, 105, 110, 115, and 120 dB at the right side and 85, 100, 110, 115, and 115 dB at the left side for stimulation at 0.25, 0.5, 1, 2, and 4 kHz frequencies, respectively (**Figure 1A**). The computed tomography (CT scan) of the temporal bone revealed normal medial and inner ear. Surprisingly, contrary to the older results the classical bi-thermal caloric stimulation with water at 30 and 44°C at adulthood (age range: 30–35 years) revealed symmetrical, good responses. The cochleography measurements were negative where EABR showed responses at stimulation with 1 mA on the right side. Consequently, the patient received CI on the right side and after the CI surgery, the PTA of implanted side reached between 25 and 35 dB across tested frequencies.

Due to a family history of hearing loss the second patient (II,1; age range: 35–40 years; a sibling to the index patient) was sent to an audiologist soon after birth (age range: 0–1 year). Comparatively, the PTA test in the childhood (age range: 0–5 years) revealed the hearing thresholds at 50, 70, 90, 100, and 100 dB for the right ear and 50, 65, 80, 90, and 110 dB on the left side at 0.25, 0.5, 1, 2, and 4 kHz frequency range, respectively. The hearing progressively deteriorated in the adulthood (age range: 25–30 years) the PTA thresholds dropped to 85, 100, 110, 115, and 120 dB for the right side and 85, 95, 120, 120, and 120 dB for the left side at 0.25, 0.5, 1, 2, and 4 kHz frequency range, respectively (**Figure 1B**). The BERA potentials were not detected even at stimulation with 110 dB click. On the other hand, the response of vestibular organs to caloric stimulation was well detected and symmetrical. The patient was fitted with bilateral hearing aids at the first year of age. The speech development was not hindered and the second patient completed regular educational program. The hearing further deteriorated to the level where no benefit from conventional hearing aids was detected (age range:25–30 years). The CT scan of temporal bones was normal. The cochleography measurements were negative where the EABR showed responses at stimulation with 300 μA on the right side and at 400 μA on the left side. After CI (age range: 25–30 years) the PTA on implanted side detected the hearing level of 30 dB through the whole frequency range.

No syndromic hearing loss indices or middle-ear related causes of hearing loss were identified in both patients. Air-bone gap did not exceed 5 dB. Additionally, both parents had normal hearing and all possible known external causes of hearing loss were excluded in both siblings.

Whole blood EDTA samples were collected for isolation of genomic DNA according to established laboratory protocols with FlexiGene DNA isolation kit (Qiagen, Hilden, Germany) (Kovač et al., 2014), to identify the underlying genetic cause of hearing loss in both siblings. Testing for the most common genetic causes of hearing loss in the population of origin – the GJB2 and TMPRSS3 variants (Battelino et al., 2015), was negative, thus qualifying both siblings for next-generation sequencing (NGS) analysis. The NGS library was prepared using TruSight One sequencing panel (Illumina, San Diego, CA, United States) according to manufacturer’s instructions. The loading concentration of prepared NGS library was 12 pM. MiSeq desktop sequencer together with MiSeq Reagent kit v3 (both Illumina, San Diego, CA, United States) were used for data collection followed by on-board primary analysis. Genetic variants with coverage >15× were analyzed with Variant Studio 2.2 software (Illumina, San Diego, CA, United States). All coding variants located outside of 100 genes related to hearing loss, reported in Hereditary Hearing Loss Homepage, were excluded from further analysis. The minor allele frequency threshold for known variants was set at 1% and all variants exceeding this value were excluded from further analysis as well.

**FIGURE 2:**
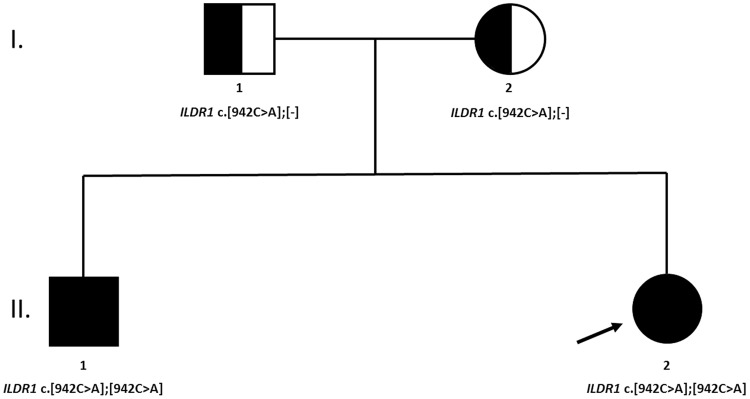
A family tree of affected family. Both parents were heterozygous carriers of the causative genetic variant. No clear indication of consanguinity was reported.

The NGS analysis revealed that both siblings were carriers of homozygous ILDR1 non-sense mutation c.942C>A (NM_001199799.1), introducing early stop codon at Cys314. The coverage of identified variant was 189× and 126× in first and second sibling, respectively. Sanger sequencing confirmed the presence of the genetic variant and its zygosity. Additionally, the family segregation analysis was performed by targeted Sanger sequencing of parental DNA samples. Both parents were carriers of a heterozygous ILDR1 c.942C>A variant (**Figure 2**).

Additional haplotype analysis of low-coverage mitochondrial genome, extracted from NGS data, was performed to obtain the insight in the ancestry of the family. Using Integrative Genomics Viewer (Thorvaldsdóttir et al., 2013) and Mitomaster (Lotti et al., 2013) tools, the mitochondrial genome haplotype of both patients was classified as J1c3a2. The identification of Y-chromosome haplotype was not successful due to too low Y-chromosome coverage in acquired NGS data.

Moreover, the ratio between rare (MAF < 1%) homozygous and heterozygous genetic variants (rare hom/het ratio) was calculated as a proxy to further assess the level of consanguinity in the family. The corresponding standard deviation score (SDS) of rare hom/het ratio was derived from comparison of patient’s ratios to a distribution of rare hom/het ratio in the Slovenian population. The population distribution of rare hom/het ratio was calculated from sequencing data of 117 anonymized individuals, generated during a routine genetic diagnostic procedure using identical abovementioned NGS library preparation protocol (TruSight One sequencing panel and MiSeq Reagent kit v3, both Illumina, San Diego, CA, United States). The coverage threshold for all variants’ datasets was set to >20×, and only variants passing this threshold were taken into a calculation of SDS of rare hom/het ratio. The SDS of rare hom/het ratio was 2.66 for the first sibling (II,2) and 1.42 for the second sibling (II,1).

## Background

Sensorineural hearing loss (SNHL) is a family of heterogeneous conditions spanning acquired as well as congenital causes of the disease. Genetic changes are one of the main contributing factors in the development of congenital SNHL. Non-syndromic hearing loss of genetic etiology has more than 100 genetic loci involved in its etiology (Parker and Bitner-Glindzicz, 2015). Additionally, more than 60 genes (Van Camp and Smith, 2017) are involved in the etiology of autosomal recessive non-syndromic hearing loss (ARNSHL), which phenotype is usually profound to severe, non-progressive and pre-lingual (Petersen and Willems, 2006). The most common genetic cause of ARNSHL are *GJB2* mutations while other reported genes include *SLC26A4*, *MYO7A*, *OTOF*, *CDH23* and *TMC1*. Interestingly, *GJB2* mutations are responsible for approximately 27% and TMPRSS3 mutations are responsible for around 13% of remaining Slovenian ARNSHL population (Battelino et al., 2015). The advent of next generation sequencing and its introduction into routine genetic diagnostic procedures accelerated the identification of causative genetic variants across heterogeneous population of patients with SNHL (Diaz-Horta et al., 2012; Yan et al., 2013).

Loss-of-function *ILDR1* mutations have been implicated in the development of very rare non-syndromic autosomal recessive deafness type 42 (DFNB42) in humans (Borck et al., 2011). *ILDR1* gene encoding the immunoglobulin-like domain containing receptor 1 is associated with the development of semicircular canal, tricellular tight junction and auditory hair cells in zebrafish and mouse models (Higashi et al., 2013; Morozko et al., 2014; Sang et al., 2014). Moreover the functional characterization of identified *ILDR1* variants in mouse mammary epithelial EpH4 cell lines revealed the disruption of tricellulin (a component of tricellular tight junction) recruitment by ILDR1 and failure to form tight junction (Kim et al., 2015).

## Discussion

A detailed clinical phenotype of two siblings with subsequently identified loss-of-function *ILDR1* mutation, from a family of European ancestry with profound hearing loss, treated with CI, is presented (**Figure 2**). The non-sense homozygous mutation in *ILDR1* gene inherited from both parents was identified as the cause of their disability. Classified as DFNB42 hearing loss, it is a very rare disorder reported in families of Pakistani, Saudi Arabian, Turkish and Iranian origin (Borck et al., 2011; Ramzan et al., 2014; Bademci et al., 2015; Mehrjoo et al., 2015) and to our knowledge this is the first report of its occurrence in the European population. The identified genetic variant *ILDR1* c.942C>A introduces early stop codon p.Cys314Ter and is reported in Human Genome Mutation Database as a disease causing variant (CM163808). It probably renders the ability of *ILDR1* to recruit tricellulin and effectively form tight junctions inefficient, consequently affecting the function of auditory hair cells as shown in animal models and cellular cultures (Morozko et al., 2014; Sang et al., 2014; Kim et al., 2015). Genetic variant *ILDR1* c.942C>A was previously reported as causative in three families of Turkish origin indicating the potential Middle-Eastern origin of the mutation (Bademci et al., 2015). The minor allele frequency of identified variant in Exome Aggregation Consortium database^1^ is 1/60642 classifying it as a very rare variant.

Analyzing the PTA data, the characteristic curves with more prominent hearing loss in higher frequency range were revealed (**Figure 1**) coinciding with previous reports (Kim et al., 2015) although there are some reports where “flat” audiograms are reported as well (Borck et al., 2011) indicating potentially diverse clinical phenotype of DFNB42. Moreover, contrary to the commonly reported phenotype (Borck et al., 2011) both patients presented with progressive and post-lingual hearing loss with the most rapid progression in the lower frequency range (**Figure 1**), supporting phenotype diversity of DFNB42 clinical manifestation. Additionally, a recent report on *ILDR1*-knockout mouse models demonstrated the progressive degradation of outer hair cells and organ of Corti, further supporting the progressive hearing loss disorder phenotype (Aslam et al., 2005; Sang et al., 2015). Nevertheless, both patients successfully finished their education program benefiting from the support of hearing aids and CI and successfully established their professional careers thereafter. The CI rescued the hearing of patients, restoring hearing in whole frequency range (**Figure 1**) to approximately 30 dB and drastically improved patients’ quality of life although there is a report where CI in *ILDR1*-misense-mutation dependent hearing loss was not successful (Kim et al., 2015). Nevertheless, when performed without clinical complications the CI is the clinical procedure of choice for optimal recovery of hearing in patients with *ILDR1*-dependent hearing loss. Additionally, the potential recovery of vestibular activation in the first patient was noticed. First caloric stimulation test of vestibular organ at the age of three was negative but the repeated test before the CI (27 years later) revealed that the vestibular response recovered. The medical records were carefully reanalyzed and the possibility of a potential analytical error was excluded. Although the existence of potential underlying recovery mechanism is a speculation, there are reports, which indicate that, at least in mice, the recovery of vestibular function could be guided through *ILDR1*-loss-of-function dependent angulin-1 mediated recovery of tricellulin localization. However, tricellulin/angulin-1 interaction does not recover hearing in mice and consequently the potential positive effect on vestibular function has to be further investigated (Higashi et al., 2015).

Consanguinity is a commonly reported underlying characteristic of *ILDR1*-dependent hearing loss resulting in a homozygous function-damaging variant segregating in affected family members (Borck et al., 2011; Ramzan et al., 2014), nevertheless, the self-reported data regarding the consanguinity in the a patient’s family didn’t indicate its possibility. But relatively high SDS of rare hom/het ratio (2.66 and 1.42, respectively) implies that at least a distant consanguinity may be a reasonable assumption. The difference between SDS of both siblings may look relatively distinct, but it translates in to the nominal difference of 1.93% (8.29% vs. 6.34%, respectively). The inter-sibling discrepancy of rare hom/het ratio SDS may originate from NGS data and library preparation alone, as both samples were not sequenced with the same quality and coverage of specific genomic regions. Consequently, this empirical evaluation may not be very precise and should be taken with a degree of reticence to use it as definite indicator of consanguinity. More accurate mapping of parental homozygous regions could not be performed as parents gave consent only to be tested for identified causative genetic variant in *ILDR1* gene. The haplotype analysis of low-coverage mtDNA revealed that both siblings are carriers of J1c3a2 haplotype. The mitochondrial haplogroup J is widespread in Europe as well as Near East. It is assumed that it was introduced into European population through Neolithic and/or Late Glacial migrations (Pala et al., 2012). Consequently, the origin of the family was established only on self-reported data from both patients and their parents.

## Concluding Remarks

This analysis confirmed the underlying variability of clinical phenotype in *ILDR1*-dependent hearing loss (Kim et al., 2015), and additionally supported the possibility of DFNB42 being a progressive hearing loss disorder (Kim et al., 2015; Sang et al., 2015), successfully treatable by CI. Additionally, this case clearly demonstrated the advantage of NGS technology in diagnostic algorithms of orphan diseases such as DFNB42. Using targeted Sanger sequencing and following the reductionist principle of genetic diagnosis where the most common causes of specific disease are tested first, the identification of rare, sporadic mutations in genes not typical for a particular population would require much more time and resources, influencing the genetic diagnosis outcome as well as the patient’s quality of life.

## Author Contributions

GK prepared the NGS libraries and performed sequencing. KP performed validation of genetic results, supervised the genetic diagnostic procedures and contributed to the manuscript. SB performed clinical evaluation of the family, performed cochlear implant surgery and contributed to the manuscript. JK analyzed NGS data, performed Sanger sequencing and wrote the manuscript.

## Conflict of Interest Statement

The authors declare that the research was conducted in the absence of any commercial or financial relationships that could be construed as a potential conflict of interest.

## Footnotes

1 http://exac.broadinstitute.org
